# Cocktail effects of clothianidin and imidacloprid in zebrafish embryonic development, with high and low concentrations of mixtures

**DOI:** 10.3389/ftox.2024.1464069

**Published:** 2024-09-18

**Authors:** Seonggeun Zee, Moonjung Hyun, Hee-Jung Sim, Kanghee Kim, Ju-Chan Kang, Chang-Beom Park

**Affiliations:** ^1^ Environmental Exposure and Toxicology Research Center, Korea Institute of Toxicology (KIT), Jinju, Republic of Korea; ^2^ Department of Aquatic Life Medicine, Pukyong National University Graduate School, Busan, Republic of Korea; ^3^ Food Safety Risk Assessment Division, National Institute of Food and Drug Safety Evaluation, Cheongju, Republic of Korea; ^4^ Bioenvironmental Science and Toxicology Division, Gyeongnam Branch Institute, Korea Institute of Toxicology (KIT), Jinju, Republic of Korea

**Keywords:** Clothianidin, Imidacloprid, binary mixtures, developmental toxicity, zebrafish embryos

## Abstract

There is growing concern that sprayed neonicotinoid pesticides (*neonics*) persist in mixed forms in the environmental soil and water systems, and these concerns stem from reports of increase in both the detection frequency and concentration of these pollutants. To confirm the toxic effects of *neonics*, we conducted toxicity tests on two *neonics*, clothianidin (CLO) and imidacloprid (IMD), in embryos of zebrafish. Toxicity tests were performed with two different types of mixtures: potential mixture compounds and realistic mixture compounds. Potential mixtures of CLO and IMD exhibited synergistic effects, in a dose-dependent manner, in zebrafish embryonic toxicity. Realistic mixture toxicity tests that are reflecting the toxic effects of mixture in the aquatic environment were conducted with zebrafish embryos. The toxicity of the CLO and IMD mixture at environmentally-relevant concentrations was confirmed by the alteration of the transcriptional levels of target genes, such as cell damage linked to oxidative stress response and thyroid hormone synthesis related to zebrafish embryonic development. Consequently, the findings of this study can be considered a strategy for examining mixture toxicity in the range of detected environmental concentrations. In particular, our results will be useful in explaining the mode of toxic action of chemical mixtures following short-term exposure. Finally, the toxicity information of CLO and IMD mixtures will be applied for the agricultural environment, as a part of chemical regulation guideline for the use and production of pesticides.

## 1 Introduction

Neonicotinoid pesticides (*neonics*) strongly bind to postsynaptic nicotinic acetylcholine receptors (*nAChRs*), which are mainly distributed in the central nervous system of insects, resulting in a variety of neurotoxic symptoms, such as convulsions, paralysis, and ultimately death (Casida and Durkin, 2013; Morrissey et al., 2015; Van der Sluijs et al., 2015). Because of the effectiveness of *neonics* against insect pests, the production and use of *neonics* have continuously increased to the point that they are the most widely used pesticides in the agricultural environment (Douglas and Tooker, 2015; Simon-Delso et al., 2015; Sparks, 2013). However, with increasing application of *neonics* in cultivated land, the environmental threat has also increased, and the concentration of *neonics* in soil has been reported to range from 0.4 to 13.28 ng/g in monitoring samples from North America and Europe (Wood and Goulson, 2017). With an increase in the concentration and toxic effects of *neonics*, which are considered emerging pollutants, the European Union (EU) and the United States have imposed sanctions to curtail agricultural applications of *neonics*, with the aim of reducing the health risks stemming from contamination in soil- and aquatic environments (European Food Safety Authority, 2013; United Stated Environmental Protection Agency, 2020). However, because there are still no regulations for environmental exposure to *neonics*, their production and use have continued in several East Asian countries (Chen et al., 2019; Christen et al., 2018). Furthermore, the sprayed *neonics* can migrate from the terrestrial to aquatic environments via natural routes, such as rainfall, meltwater, and polluted dust. Indeed, *neonics* have been detected in surface water, at concentration ranging from 0.13 to 0.63 ng/mL (Botías et al., 2015; Hladik and Kolpin, 2016; Limay-Rios et al., 2016; Main et al., 2016). Thus, to clearly understand the eco-health risks of *neonics* used in agricultural, an assessment of the toxic effects of *neonics* in both terrestrial- and aquatic environments is required.

Clothianidin (CLO) and imidacloprid (IMD) are believed to be the most widely used *neonics* worldwide (Miles et al., 2017; Woodward et al., 2022). CLO and IMD can be directly introduced into the terrestrial environment during the application season before the harvest of agricultural products (Struger et al., 2017), and their release at high concentrations into aquatic environments is possible due to their high solubility and slow rate of chemical decomposition (Morrissey et al., 2015). For example, in soil samples from agricultural land in the United States, CLO at concentrations of 0.02–13.6 ng/g and IMD at 0.09–10.7 ng/g were detected at the time of application (Jones et al., 2014). In term of water pollution, concentrations of *neonics* as high as 59–61 ng/L (CLO) and 70–149 ng/L (IMD) have been found in water samples from vicinity of wastewater treatment plants in the United States (Sadaria et al., 2016).

Generally, CLO and IMD residues that accumulated on the plant surface or soils after spraying on agricultural environment can mainly transported into the surface water by precipitation or runoff. Interestingly, CLO and IMD were detected in 100% of the runoff samples from agricultural environment in the Salinas Valley, California, simultaneously. The detected concentrations were varied 32–576 ng/L for CLO and 11–274 ng/L for IMD after seed treatment and their concentrations were elevated from 4,877 ng/L in runoff samples after drench treatment (Woodward et al., 2022). These reports indicate that CLO and IMD residues are existed the form of chemical mixture in soil and aquatic environment. Indeed, the measured concentrations of CLO and IMD in the mixed form were approximately 1:1 in soil and aquatic environments (Bonmatin et al., 2015; Sánchez-Bayo et al., 2016). These results are not irrelevant the increase of health risks of co-exposure to CLO and IMD in both soil and aquatic environment (Sánchez-Bayo et al., 2016; Simon-Delso et al., 2015). For this reason, the toxic effects of pesticide mixtures, including *neonics* mixtures, have become an important issue in the field of environmental health. In previously studies, acute and chronic exposure to different combinations of CLO and IMD has been shown to have a dose-dependent synergistic toxic effect on bloodworm larvae (Maloney et al., 2017; 2018). However, although aquatic organisms may be directly exposed to a mixture of CLO and IMD at high concentrations during the pesticide spraying period, the information of toxicity caused by the combination of CLO and IMD at environmental concentrations is insufficient.

The objective of this study is to assess the mixture toxicity of CLO and IMD at environmental concentrations reflecting both low and high concentrations (e.g., from ng/mL to μg/mL) during the intensive spraying season in agricultural lands. To confirm the mixture toxicity of CLO and IMD at environmental concentrations, zebrafish embryos were selected as test model organisms. Because developmental defects caused by exposure to mixtures of CLO and IMD in zebrafish embryos indicate non-negligible ecological risks at the individual and the population level (Embry et al., 2010). To ascertain the developmental toxicity of mixtures of CLO and IMD, which are dependent on exposure levels (e.g., mixed concentrations), the toxicity tests using zebrafish embryos were conducted with mixtures of CLO and IMD at different exposure levels through the intrinsic toxicity of a single chemical. In this study, we report differences in toxicity between mixture compounds that is potential mixtures based on the effective concentration of a single chemical and realistic mixtures, which are based on actual measured environmental concentrations. The potential toxic effects in zebrafish embryonic development were quantified in terms of the degree of alterations of target genes expression, namely cell damage and thyroids imbalance.

## 2 Material and methods

### 2.1 Target chemicals

Clothianidin (CLO, CAS no. 210880-92-5, >98%) and imidacloprid (IMD, CAS no. 138261-41-3, >98%) were purchased from Sigma-Aldrich (St. Louis, MO, United States). Stock solutions were prepared in dimethyl sulfoxide (DMSO; Sigma-Aldrich, St. Louis, MO, United States). ≤ 1% (v/v) DMSO was used as solvent control for the toxicity tests. Because there was not significant different between the developmental toxicity of zebrafish, including the activation of stress response, when compared to 0.1% DMSO exposed group with the control group through the previous study (Xiong et al., 2017).

### 2.2 Single toxicity tests for zebrafish embryos

Zebrafish embryos were obtained from adult zebrafish continuously sub-cultured in Korea Institute Toxicology Laboratory, Republic of Korea and were maintained at 26°C ± 1°C in a climate chamber (16 h daylight: 8 h darkness) until use for toxicity tests (Park et al., 2022). To confirm the intrinsic toxicity of CLO and IMD in zebrafish embryos, 20 zebrafish embryos in each exposure group were used for single toxicity tests at nine nominal concentrations [0 (DMSO >0.1%), 7.8, 15.6, 31.3, 62.5, 125, 250, 500, and 1,000 μg/mL] in a sterilized cell culture 6-well plate filled with 15 mL working solution (Effendorf, Hamburg, Germany). At 6 days after exposure, embryonic toxicity (%) was scored as follows: [embryonic toxicity (%) = lethality (dead embryo + dead larva) + abnormality (unhatched eggs + abnormal larva)/initial embryos × 100]. Developmental abnormality of zebrafish was investigated based on apical phenotypic observation, such as coagulated embryos, lack of somite formation, non-detachment of the tail, and lack of heartbeat for 6 days after exposure (OECD, 2013). Embryonic toxicity (%) was used for estimating the effective concentration values (EC*x*) for each chemical and 95% confidence limits, by concentration-response curves (CETIS program version 1.8.7.15, Tidepool Scientific Software, United States) (Park et al., 2022).

### 2.3 Mixture toxicity tests for zebrafish embryos

Based on the intrinsic toxicity levels of each chemical in and zebrafish embryos, mixture toxicity tests were conducted with two different mixture compounds: a potential mixture compounds that can occur high concentrations during pesticide spraying period or long-term exposure, and realistic mixture compounds that reflect environmental concentrations.

All toxicity tests were approved by the Institutional Animal Care and Use Committee (IACUC) in Korea Institute of Toxicology, Republic of Korea (IACUC no. 2006-0003).

#### 2.3.1 Potential mixture toxicity tests

Both CLO and IMD, individually, were found to be toxic to zebrafish embryos at high concentrations, namely 500 and 1,000 μg/mL (Supplementary Figure S1). Potential mixture toxicity tests for zebrafish embryos were designed with EC*x* values estimated from a single toxicity test (Table 1; Supplementary Figure S1), which involved a range from the lowest effective concentration (EC5) to the half effective concentration (EC50) for each target chemical. Potential mixture toxicity tests in the embryonic stages of zebrafish were performed under the same conditions as the single toxicity tests, and the mixing ratios of CLO and IMD were fixed at 1:1, reflecting the detected mixing ratio in the natural environment (Bonmatin et al., 2015; Sánchez-Bayo et al., 2016).

**TABLE 1:** Effective concentrations (EC*x*) of clothianidian (CLO) and imidacloprid (IMD) in zebrafish at the embryo-larva stage after exposure for 6 days. The 95% confidence limits (95% CI) were calculated using the probit method (CETIS program version 1.8.7.15, Tidepool Scientific Software, United States).

Toxicity test	EC5 (95% CI)	EC15 (95% CI)	EC25 (95% CI)	EC50 (95% CI)
CLO (µg/mL)	65.28 (N.C.–104.61)	255.72 (224.61–289.12)	292.89 (247.77–332.07)	411.11 (345.75–535.54)
IMD (µg/mL)	60.80 (N.C.–108.36)	145.69 (113.89–158.99)	175.04 (146.25–196.54)	267.96 (205.83–294.60)

N.C., no calculation.

#### 2.3.2 Realistic mixture toxicity tests

To verify the developmental effects in zebrafish embryos caused by exposure to realistic mixtures, mixture toxicity tests with zebrafish embryos were performed at a range of environmentally relevant concentrations, and three exposure concentrations at a 1:1 mixing ratio (i.e., 1, 10, and 100 ng/mL of each chemical). The exposure concentrations and mixing ratios of CLO and IMD in the mixtures reflected actual measured environmental concentrations (Bonmatin et al., 2015; Sánchez-Bayo et al., 2016). In addition, to examine the toxicity pathway related to embryonic development, zebrafish embryos exposed to mixtures of CLO and IMD were sampled at 6 days after exposure and placed in a sterilized 1.5 mL microtube with 100 μL of RNAlater™ (Thermo Fisher Scientific, Massachusetts, United States) and were stored at −40°C until use in the expression change analysis of target genes. Realistic mixture toxicity tests using zebrafish embryos were performed under the same conditions as the single-toxicity tests.

#### 2.3.3 Residual concentrations of CLO and IMD in the test media

The concentrations of CLO and IMD in the zebrafish test media were analyzed using high performance liquid chromatography–tandem mass spectrometry (HPLC-MS/MS) with an Agilent 6,420 Triple Quadrupole MS instrument connected to an Agilent 1,260 Infinity HPLC system containing a binary pump, autosampler, and degasser (Agilent, Santa Clara, CA, United States). Chromatographic separation was performed using a Poroshell 120 EC-C18 column (2.1 × 100 mm, 2.7 µm particle size; Agilent, Santa Clara, CA, United States) connected to a C18 guard column (Phenomenex, Torrance, CA, United States). Mobile phase A was 0.1% formic acid in water, and mobile phase B was acetonitrile. HPLC was run using a linear gradient as follows: 0–5 min, 20%–40% B; 5–7 min, 40%–70% B. The column was then equilibrated with 20% B for 4.9 min. The flow rate was set at 0.4 mL/min, and the injection volume was set at 10 μL. MS analysis was conducted in the positive ion mode using electrospray ionization. The MS conditions were optimized as follows: capillary voltage, 4000 V; sheath gas temperature, 370°C; sheath gas flow, 12 L/min; gas temperature, 350°C; gas flow, 8 L/min; nebulizer gas pressure, 40 psi; fragmentor voltage, 75 V for CLO and 80 V for IMD. On the selected reaction monitoring mode (SRM), the collision energy (CE) and selected reaction monitoring channels for the analytes were as follows: CLO, CE 10 V and *m/z* 250.0→169.0; CLO-d3, CE 10 V and *m/z* 253.0→172.0; IMD, CE 12 V and *m/z* 256.0→209.0; IMD-d4, CE 10 V and *m/z* 260.1→213.1. HPLC-MS/MS data were collected and processed using MassHunter Workstation Software Qualitative Analysis (ver. B.06.00, Agilent, Santa Clara, CA, United States). The relative error (RE) values for the residual concentration of each target chemical were calculated as: RE (%) = (mean concentration of media samples–nominal concentration)/nominal concentration) × 100).

#### 2.3.4 Transcriptional alteration of target genes

To examine the transcriptional alteration of target genes linked to zebrafish embryonic development, The total RNA from 20 zebrafish embryos was extracted using a RNeasy Mini kit (Qiagen, Hilden, Germany), following the manufacturer’s instructions. The total RNA concentration was measured spectrophoto-metrically at 260/280 nm using a Gen5™ spectrophotometer (BioTek^®^, Winooski, VT, United States). First-strand cDNA was synthesized with 0.5 µg of total RNA using a Superscript III First-Strand Synthesis System kit (Thermo Fisher Scientific, Vilnius, Lithuania) according to the manufacturer’s manual.

The primer sequences for target genes were designed to use for quantitative real-time polymerase chain reaction (qRT–PCR): namely aryl hydrocarbon receptor 2 (*ahr2*), cytochrome P450 monooxygenases (i.e., *cyp1a1* and *cyp1b1*), nuclear factor erythroid 2-related factors (i.e., *nrf1a* and *nrf2a*), tumor suppressor gene (*p53*), thyroid stimulating hormone beta subunit (*tsh-β*), and thyroid hormone receptors (i.e., *thraa* and *thrb*) (Supplementary Table S1) (Fan et al., 2018; Liu et al., 2011; Ren et al., 2020; Williams et al., 2013). The qRT–PCR was performed using a Thermal Cycler Dice^®^ Real Time System III (Takara, Tokyo, Japan) using the Go Taq^®^ qPCR Master Mix (Promega, Madison, WI, United States). The qRT–PCR reactions were conducted as follows: an initial hot-start activation step at 95°C for 2 min, followed by 40 cycles at 95°C for 15 s and 60°C for 60 s. Melting curve analyses were performed to optimize primers for qRT–PCR performance. These transcript abundances for the target genes were normalized to a housekeeping gene (i.e., *β-actin*). The relative quantification of target genes expression was calculated using the 2^−ΔΔCT^ method (Park et al., 2022).

### 2.4 Statistical analysis

All data are represented as mean ± standard error of the mean (SE). Data comparisons between the exposure groups in the toxicity tests were conducted using the *post hoc* least squares distance (LSD) test method of one-way analysis of variance (ANOVA) (SigmaPlot version 12.5; Systat Software, Inc., San Jose, CA, United States). *P*-values less than 0.05 (*P* < 0.05) were considered statistically significant.

To determine the combined effect for binary mixtures of CLO and IMD in zebrafish embryo toxicity tests, we used the combination index (CI) equation, which is given by CI = (D)_1_/(D*x*)_1_ + (D)_2_/(D*x*)_2_ for a combination of two substances; where (D*x*)_1_ and (D*x*)_2_ in the denominator are the dose of each substance “alone” that provides *x* % effect (EC*x*), and (D)_1_ and (D)_2_ in the numerator are the dose of each substance in the mixture that provides *x* % effect (Chou, 2011). The CI value was calculated with regard to CompuSyn software (ComboSyn, Inc., NJ, United States), and the isobologram was plotted using sigmaplot software (version 15.0, Systat Software, Inc., Canada).

## 3 Results and discussion

In this study, we hypothesized that exposure to a mixture of CLO and IMD could induce developmental toxicity in aquatic organisms through cumulative exposure to low concentrations (non-lethality); i.e., the lowest observed effect concentration. To verify our hypothesis, binary mixture toxicity tests for CLO and IMD were performed with zebrafish embryos under mixture combinations from half effective concentrations for each chemical (potential mixture toxicity) to environmental concentrations (realistic mixture toxicity).

### 3.1 Potential mixture toxicity of CLO and IMD

**FIGURE 1:**
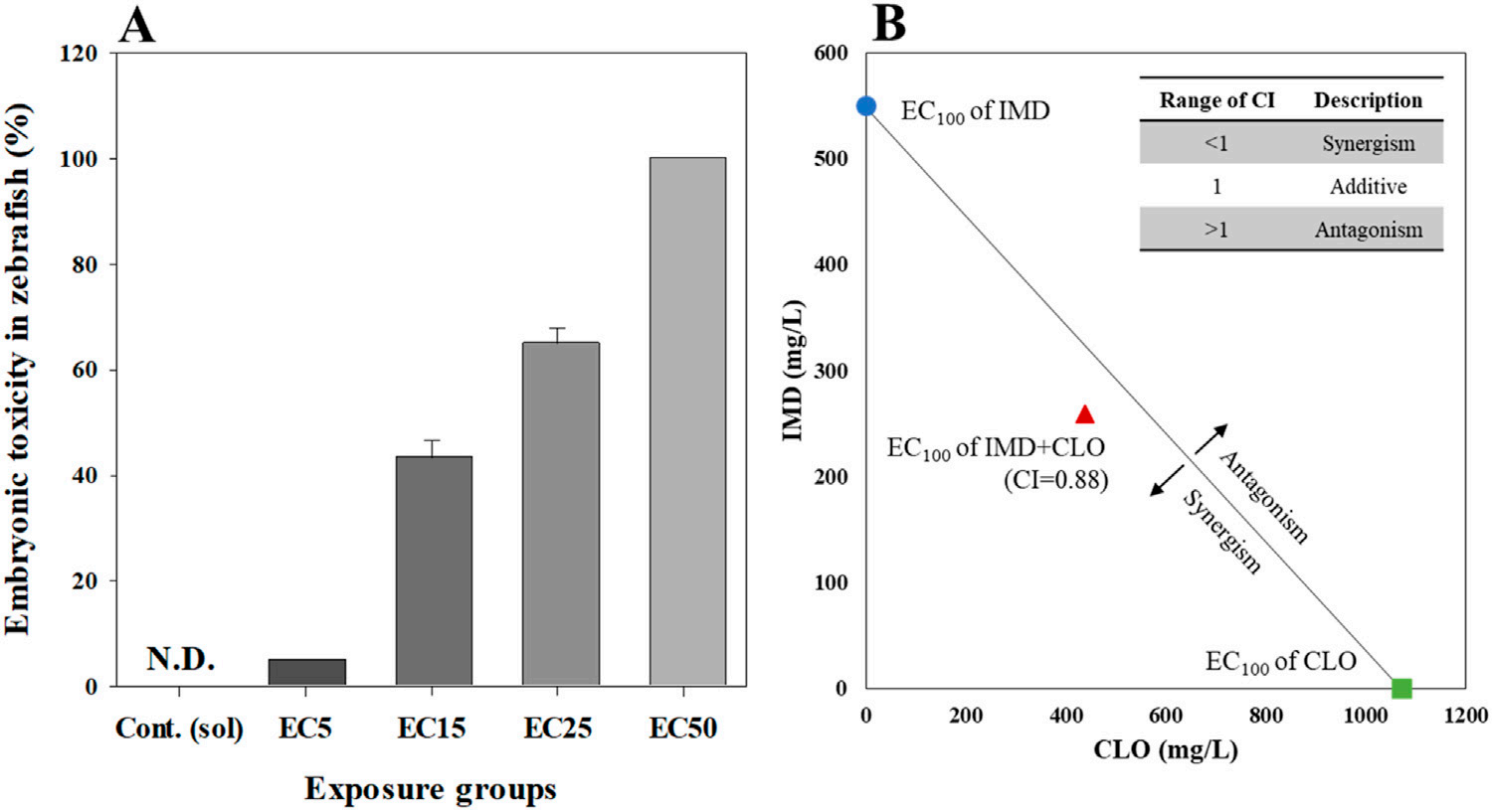
Embryonic toxicity **(A)** and combination index (CI) value **(B)** for binary mixtures of clothianidin (CLO) and imidacloprid (IMD) in zebrafish at 6 days after exposure. Zebrafish toxicity tests were designed with EC*x* values estimated from single toxicity: the mixture included from 5% to 50% effective concentrations of each of the two chemicals, and the mixing ratio was 1:1. All data represent the mean value ±standard error (SEM) of triplicate experiments.

In binary mixture toxicity tests, owing to the effective concentrations of CLO and IMD, the combined effects of CLO and IMD on embryonic toxicity development in zebrafish were observed. In potential mixture toxicity tests, the mixtures of CLO and IMD based on EC*x* values for each chemical (i.e., 60–400 μg/mL of CLO and IMD) showed higher toxicity than single chemical exposure, and elevated with exposure concentrations (Figure 1A). To confirm the combined effect for binary mixtures of CLO and IMD, the concept of CI equation was introduced based on the median-effect equation of the law of mass action; where CI = 1, <1, and >1 indicate additive effect, synergism, and antagonism, respectively (Chou, 2011). In this study, CI value for binary mixture of CLO and IMD values was <1, and these results indicated that binary mixtures of CLO and IMD have synergistic effect in zebrafish embryonic toxicity with a dose-dependent manner (Figure 1B) (Maloney et al., 2017; 2018). Our results confirmed that combinations of potential toxicity test of mixtures may amplify the toxic effects of single CLO and IMD those were observed. Therefore, we should consider the toxic effects of both individual pesticides and their mixtures in the risk management of pesticides for protecting the soli and aquatic ecosystems (Wu et al., 2018).

We, also, confirmed that IMD may act as an accelerator of toxic effects in binary mixtures of CLO and IMD, due to that IMD exposure showed a strong toxicity when compared to CLO exposure in this study: EC50 values were 411.11 μg/mL for CLO and 267.96 μg/mL for IMD, respectively (Table 1). In a study of CLO and IMD acute toxicity in sheepshead minnow (Gibbons et al., 2015), it was found that IMD triggered mixture toxicity due to higher toxicity of IMD than that of CLO; the 50% lethal concentration (LC50) was 161 μg/mL for CLO and 93.6 μg/mL for IMD. However, it is difficult to determine whether IMD is the main toxic compound in the CLO and IMD mixtures from the toxic units (i.e., EC*x*) for zebrafish embryos in potential mixture toxicity tests. Mixture toxicity tests under different mixture combinations (e.g., ratios and concentrations) are required to clearly identify the toxic compounds in the mixtures. Consequently, our findings indicate that toxicity tests for zebrafish embryos are appropriate for applying the combined effects of CLO and IMD in natural environments. In this study, realistic mixture toxicity tests were conducted with zebrafish embryos, to confirm the adverse effects of mixtures at environmental concentration of CLO and IMD.

### 3.2 Realistic mixture toxicity of CLO and IMD

#### 3.2.1 Residual concentrations of CLO and IMD

**FIGURE 2:**
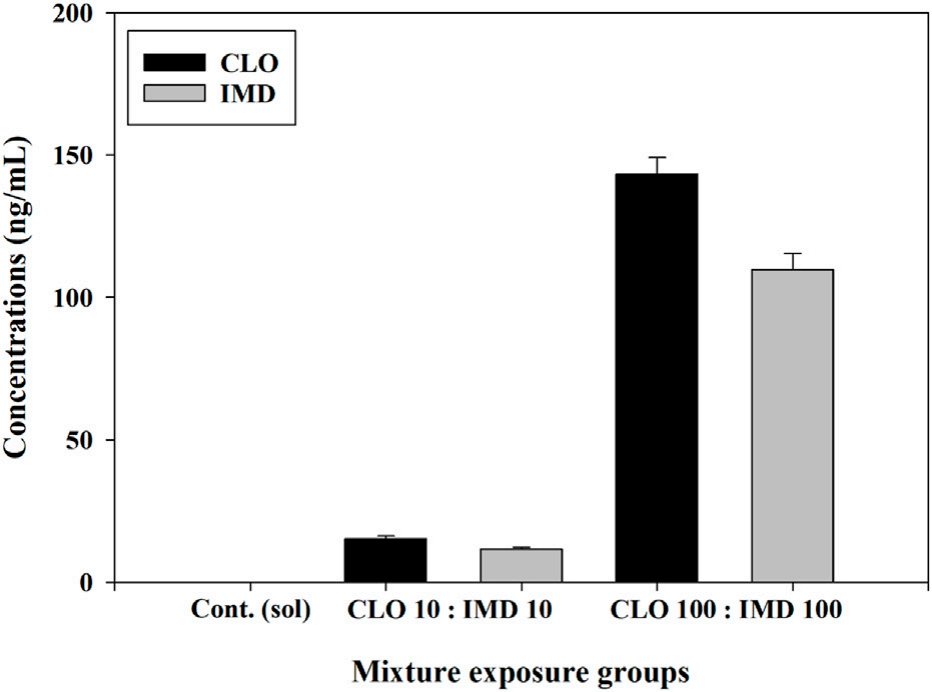
Residual concentrations of clothianidin (CLO) and imidacloprid (IMD) in binary mixtures for zebrafish embryos. The media combined with CLO and IMD at high and low concentrations (i.e., 10 and 100 ng/mL) were analyzed using the HPLC-MS/MS method in triplicate experiments. Seven-point calibration curves were generated from 2 to 500 ng/mL with an R^2^ value >0.999, and the recovery test of this method was conducted at three concentrations (5, 75, and 400 ng/L).

The stability and persistence of CLO and IMD in binary mixtures were assessed through two different types of mixing combinations, high and low concentrations (i.e., mixtures containing 10 and 100 ng/mL of each chemical, respectively), at 6 days after exposure. CLO and IMD were not detected in the control solution. Residual concentrations (compared to initial concentrations) of CLO and IMD in the mixture solutions ranged from 92.1% to 103.0% in the case of CLO and from 91.6% to 101.7% in the case of IMD, which indicates that both of these *neonics* were stable in the mixtures; that is, no or negligible volatilization or denaturation (Figure 2). Furthermore, the half-life of CLO reaches 56 days in sediment and up to 1,386 days in soil, and the half-life of IMD reaches 129 days in sediment and up to 228 days in soil (Mason et al., 2013). These results indicate that mixtures of CLO and IMD can steadily accumulate and persist in aquatic environments after agriculture or soil applications. These phenomena are not independent of the continuous increase in the detected concentrations of CLO and IMD in surface waters over the past 15 years and may lead to acute toxicity in aquatic organisms, as well as chronic toxicity (Sánchez-Bayo et al., 2016; Simon-Delso et al., 2015). Therefore, to better understand the toxic effects of CLO and IMD mixtures, considering their detected environmental concentrations, further studies on the phenotypic toxicity caused by short-term exposure and the toxic mechanisms for predicting chronic toxicity are required.

#### 3.2.2 Mixture toxicity reflecting the environmental concentrations

Some studies reported that CLO and IMD might be remained in high concentrations in surface waters, through leaching and runoff under worst-case environmental scenarios (e.g., incorrect handing or improper disposal): up to IMD 320 ng/mL in Netherlands (Van Dijk et al., 2013) and CLO up to 170 ng/mL in Unite States (Miles et al., 2017). Therefore, we hypothesized that single and mixture of ≤100 ng/mL CLO and IMD can reflect as the detectable concentrations in the aquatic environment, including worst-case scenarios. In the present study, phenotypic toxicity was not observed in binary mixtures of CLO and IMD that reflected the detectable concentrations in the aquatic environment. There was no significant difference between all exposure groups compared with solvent control group, indicating that embryonic toxicity on zebrafish embryos, i.e., lethality and abnormality, were less than 15% after binary mixture exposure (Table 2). Thus, when exposed to detectable concentrations in the aquatic environment (i.e., ≤100 ng/mL), CLO and IMD, whether singly or in mixtures, are little to cause short-term developmental toxicity in zebrafish embryos. However, a strong acute toxicity to zebrafish was shown in the *neonics* mixtures-exposure groups and this phenomenon was exerted by synergistic effects with mixing ratio and concentration of components in the mixtures. These results imply that *neonics* mixture toxicity was associated with co-existing components and concentrations in the mixtures (Shukla et al., 2017; Wang et al., 2017). Therefore, although there was no phenotypic toxicity in zebrafish embryos, developmental defects in zebrafish embryos may appear upon co-exposure at low concentrations by mixing ration and concentrations of CLO and IMD (Wu et al., 2018).

**TABLE 2:** Embryonic toxicity of clothianidian (CLO), imidacloprid (IMD), and binary mixtures for 6 days after exposure. Mix. 1, 10, and 100 indicate binary mixtures consisting of 1, 10, and 100 ng/mL of each two chemicals. All data represent the mean value ± standard errors (n = 20 embryos in each group with triplicate experiments).

Toxicity tests	Exposure groups (ng/mL)					
	Cont. (Sol)	CLO 10	IMD 10	Mix. 1	Mix. 10	Mix. 100
Embryonic toxicity (%)	3.3 ± 1.7	12.5 ± 3.4	12.5 ± 2.8	8.3 ± 1.7	11.7 ± 2.5	7.5 ± 1.7

In this study, because ≤10 ng/mL CLO and IMD were simultaneously detected in runoff samples after spraying on agricultural environment (Woodward et al., 2022), the combined effects of CLO and IMD on zebrafish embryonic development were compared with 10 ng/mL single chemical-exposure group as a positive control. The mixture effects of CLO and IMD at realistic environmental concentration and mixing ratio, including worst-case scenarios (i.e., ≤100 ng/mL), were assessed with the toxic alteration of two different pathways which can affect the development of zebrafish embryos: the expression levels of target genes that are induced to cell damage by oxidative stress response and involved in thyroid hormone biosynthesis.

Aryl hydrocarbon receptor-mediated oxidative stress can induce toxic signaling pathways linked to cell damage, such as irritation of the inflammatory reactions or the induction of peroxidases by cytochrome P450-dependent oxygenase, during the early stages of embryonic development (Jin et al., 2020; Ren et al., 2020; Shankar et al., 2020). Several studies reported that the activation to oxidative stress is regulated by the redox-sensitive nuclear factor erythroid 2-related factors (Rousseau et al., 2015; Ulin et al., 2019; Williams et al., 2013). Because oxidative stress response also may affect the expression of proto-oncogene (*p53*), a tumor suppressor gene that regulates the cell cycle, we targeted genes which are involved aryl hydrocarbon receptor-mediated oxidative stress response, including *p53*, to examine the mixture toxicity of CLO and IMD at environmental concentrations (Chen, 2016; Fan et al., 2018; Mugoni et al., 2014). The adverse effects caused by the form of realistic mixtures of CLO and IMD in zebrafish embryos were evaluated with the expression levels of targeted genes after binary mixtures exposure.

**FIGURE 3:**
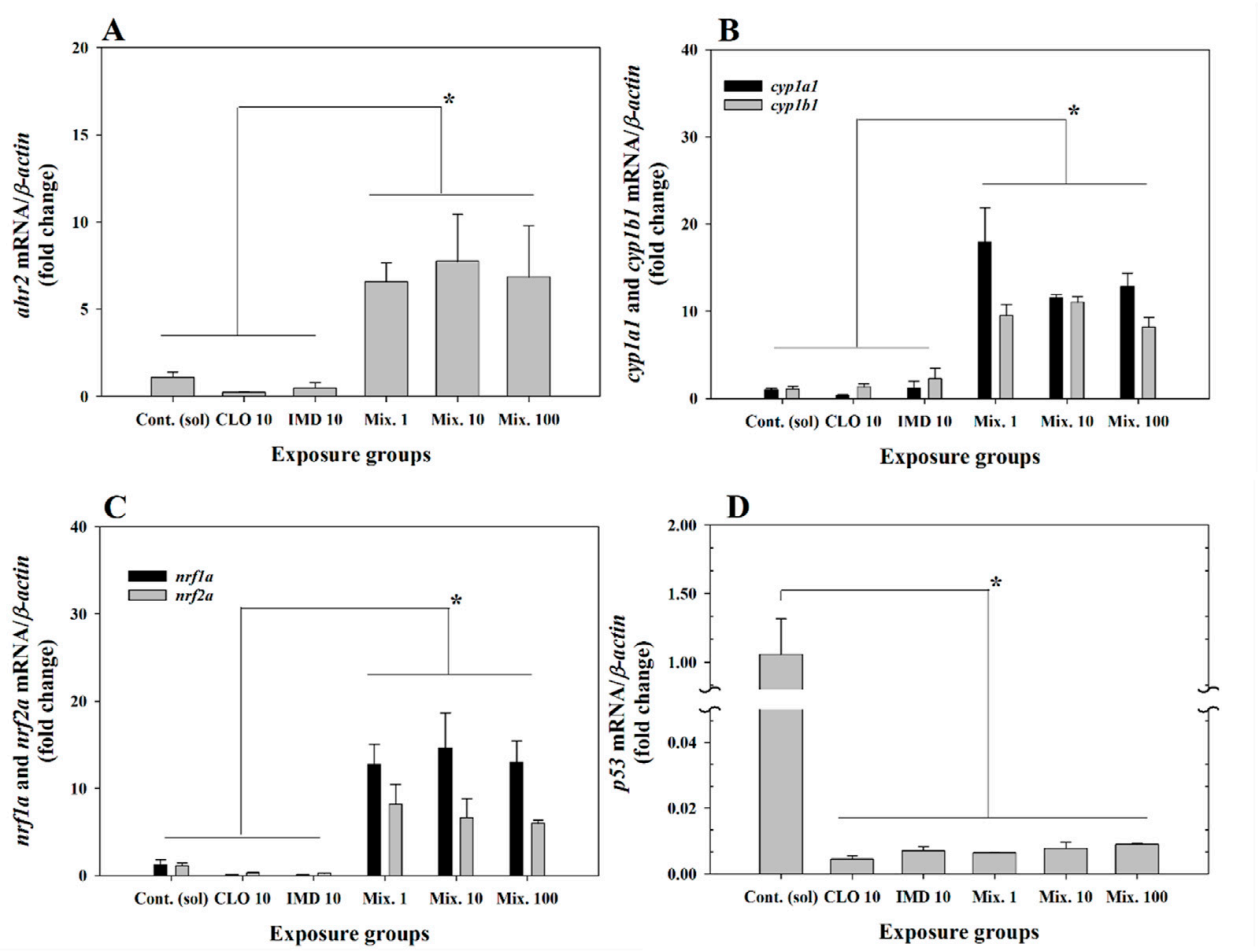
Relative fold change of the expression levels for aryl hydrocarbon receptor 2 (*ahr2*), cytochrome P450 monooxygenases (*cyp1a1* and *cyp1b1*), nuclear factor erythroid 2-related factors (*nrf1a* and *nrf2a*), and tumor suppressor gene (*p53*) in zebrafish embryos exposed to 10 ng/mL of clothianidin (CLO), 10 ng/mL of imidacloprid (IMD), and binary mixtures at three different concentrations (i.e., 1, 10, and 100 ng/mL) with a 1:1 mixing ratio: **(A)**, *ahr2*; **(B)**, *cyp1a1* and *cyp1b1*; **(C)**, *nrf1a* and *nrf2a*; **(D)**, *p53*. The expression levels of genes were normalized to *β-actin* and values are presented as the mean value ±standard error (SEM) (n = 20 embryos in each exposure groups with three replicates). *Different letters indicate significant differences between the exposed groups (*P* < 0.05).

Interestingly, the expression levels of target genes linked to the cell damage pathway [aryl hydrocarbon receptor (*ahr2*), cytochrome P450 subunits (*cyp1a1* and *cyp1b1*), nuclear factor erythroid 2-related factors (*nrf1a* and *nrf2a*), and *p53*] were markedly up- or downregulated in the mixture exposure groups compared to the corresponding gene expression levels in the control group (*P* < 0.05, Figure 3; Supplementary Table S2). These results indicate that exposure to binary mixtures of CLO and IMD may cause developmental defects in zebrafish embryos through cell damage mediated by chemical stimulation, even at low concentrations. Previously other studies were reported that the transcriptional expression of *ahr2* mRNA, which reacts to chemicals with aromatic carbocyclic rings (e.g., *neonics*), induces the transcriptional activation of cytochrome P450 monooxygenases (*cyp1a1* and cyp1b1) and nuclear factor erythroid 2-related factors (*nrf1a* and *nrf2a*), indicating that their transcriptional alterations involve cellular oxidative stress response to xenobiotics (Jin et al., 2020; Ren et al., 2020; Rousseau et al., 2015; Shankar et al., 2020; Ulin et al., 2019; Williams et al., 2013). Excessive oxidative stress downregulated the transcriptional level of *p53*, leading to cell cycle arrest and/or cytotoxicity (Chen, 2016; Fan et al., 2018; Mugoni et al., 2014). Consequently, although abnormalities were not observed in short-term toxicity tests, in the present study, alterations in the transcriptional levels of target genes for cell damage implied that exposure to binary mixtures of CLO and IMD may induce developmental defects in zebrafish embryos through DNA damage linked to acute oxidative stress responses (*P* < 0.05, Figure 3; Supplementary Table S2).

Thyroid hormones are involved in early development and growth-related biological processes and functions in zebrafish embryos (Liu and Chan, 2002). In particular, to determine the zebrafish embryonic toxicity for potentially harmful substances, thyroid stimulating hormone beta (*tsh-β*) which promotes thyroid hormone secretion and thyroid hormone receptor alpha and beta (*thraa* and *thrb*) have been used as toxicological indicators.

**FIGURE 4:**
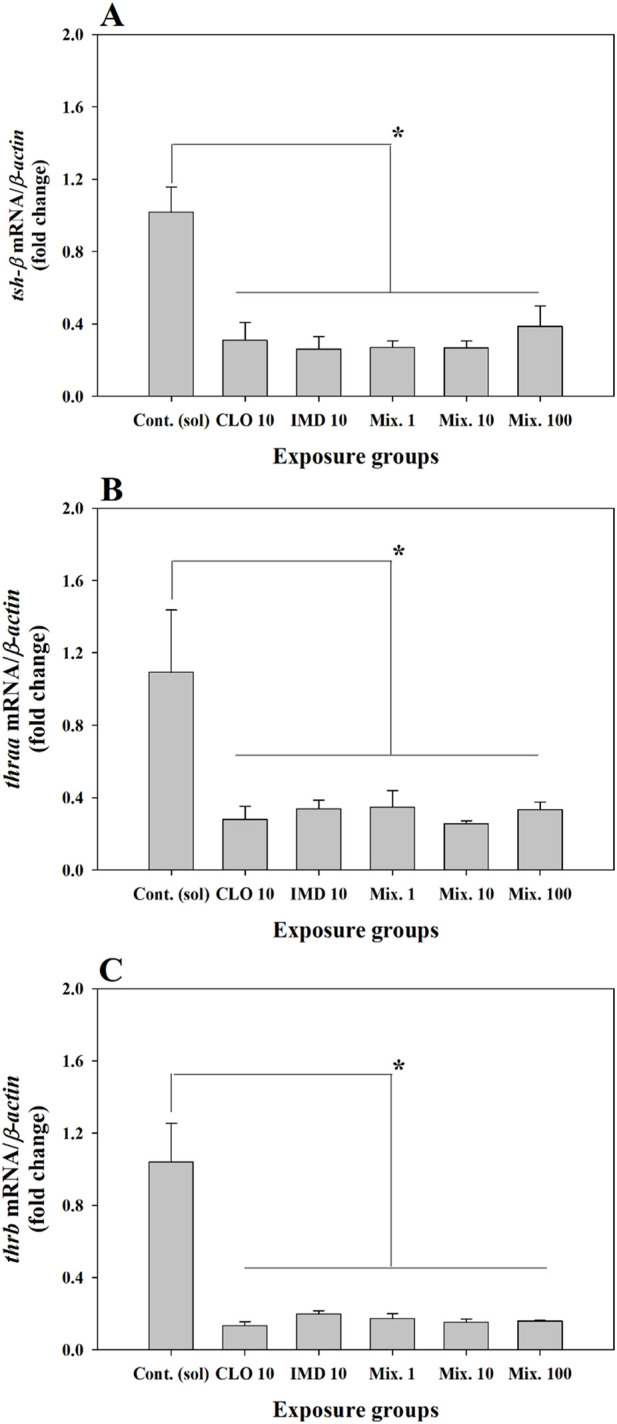
Relative fold change of the expression level for thyroid stimulating hormone beta subunit (*tsh-β*) and thyroid hormone receptors (*thraa* and *thrb*) in zebrafish embryos exposed to 10 ng/mL of clothianidin (CLO), 10 ng/mL of imidacloprid (IMD), and binary mixtures at three different concentrations (i.e., 1, 10, and 100 ng/mL) with a 1:1 mixing ratio: **(A)**, *tsh-β*; **(B, C)**, *thraa* and *thrb*. The expression levels of genes were normalized to *β-actin* and values are presented as the mean value ±standard error (SEM) (n = 20 embryos in each exposure groups with three replicates). *Different letters indicate significant differences between the exposed groups (*P* < 0.05).

In this study, the transcriptional levels of *tsh-β*, *thraa*, and *thrb* were significantly decreased in the single- and binary mixture groups of CLO and IMD compared to those in the control group (*P* < 0.05, Figure 4; Supplementary Table S2). Our findings indicate that CLO and IMD, either singly or combined in mixtures, can disturb thyroid hormone biosynthesis in the embryo-larva development of zebrafish. Similar to our findings, some studies reported that hazardous chemicals distributed in natural environment, including *neonics*, can interfere with the activation of thyroid hormones through receptor-mediated signal transduction (Boas et al., 2012; Liu et al., 2011; Ma et al., 2019; Walter et al., 2019). Thus, these results imply that the disturbance of thyroid hormone biosynthesis may be due to the intrinsic toxic characteristics of single chemicals and can be promoted by binary mixture exposure (*P* < 0.05, Figure 4; Supplementary Table S2). However, synergistic effects on thyroid hormonal activity were not confirmed in the mixtures. To more clearly elucidate the combined effects on developmental endocrine system in zebrafish, such as thyroid hormone biosynthesis, further studies examining the developmental impairment of zebrafish embryos through chemical interactions in the mixtures are required.

Taken together, a novel finding is that exposure to binary mixtures of CLO and IMD in the aquatic environment (i.e., concentration and ratio) can cause the promotion of cell damage by oxidative stress response and the suppression of thyroid hormone synthesis which is involved embryonic development. This phenomenon indicate that the expression alteration of target genes linked to cell damage and thyroid hormonal synthesis could impair the embryonic development in zebrafish, by the components and concentrations of chemical mixtures (Wang et al., 2020; Wu et al., 2018). Our results will be useful in examining the combined effects of mixtures following short-term exposure, to predict the developmental toxic effects of chronic exposure to CLO and IMD. However, to clearly confirm the developmental toxicity caused by significant changes of target genes linked to cell damage and thyroid hormonal synthesis, it is necessary to investigate the effects of long-term exposure as well as the expressions of corresponding genes.

## 4 Conclusion

We confirmed the potential for increased combined concentrations of CLO and IMD in aquatic environments owing to their high stability and continuity. Even at a low concentration reflecting the measured environmental concentration, long-term co-exposure to CLO and IMD was found to induce not only cell damage by oxidative stress response but also developmental disturbance via thyroid hormone biosynthesis disruption, resulting in developmental defects of zebrafish. Therefore, exposure to mixtures of CLO and IMD in the range of environmental concentration may pose a greater threat to zebrafish embryonic development than would exposure to either of the two chemicals. The results from this study can introduce a scientific information for safety application of CLO and IMD to chemical regulation. To provide a comprehensive insight into the combined effects and their action mechanisms of CLO and IMD at the environmental concentration level, chronic exposure to chemical compounds, including behavioral changes, growth parameters, histological pathology, and reproductive effects, is required.

## Data Availability

The datasets presented in this study can be found in online repositories. The names of the repository/repositories and accession number(s) can be found in the article/Supplementary Material.

## Abbreviations

ahr2, Aryl hydrocarbon receptor; cyp1a1 and cyp1b1, cytochrome P450 subunits; CLO, clothianidin; IMD, imidacloprid; nAChRs, nicotinic acetylcholine receptors; neonics, neonicotinoid pesticides; nrf1a and nrf2a, nuclear factor erythroid 2-related factor; p53, proto-oncogene; tsh-β, thyroid stimulating hormone beta; thraa and thrb, thyroid hormone receptor alpha and beta.
