# Supplementary material for: Cocktail effects of clothianidin and imidacloprid in zebrafish embryonic development, with high and low concentrations of mixtures
Source: Front Toxicol. 2024 Sep 18;6:1464069. doi: 10.3389/ftox.2024.1464069 (PMC11445189; doi:10.3389/ftox.2024.1464069)
Supplement: Supplementary file 1 [file DataSheet1.docx]

**Cocktail effects of clothianidin and imidacloprid** **in zebrafish embryonic development, with high and low concentrationd of mixtures**

Seonggeun Zee^a,c,d,ǂ^, Moonjung Hyun^b,ǂ^, Hee-Jung Sim^a^, Kanghee Kim^a^, Ju-Chan Kang^c^, Chang-Beom Park^a,*^

^a^Environmental Exposure & Toxicology Research Center, Korea Institute of Toxicology (KIT), Jinju 52834, Republic of Korea

^b^Bioenvironmental Science & Toxicology Division, Gyeongnam Branch Institute, Korea Institute of Toxicology (KIT), Jinju 52834, Republic of Korea

^c^Department of Aquatic Life Medicine, Pukyong National University Graduate School, Busan 48513, Republic of Korea

^d^Food Safety Risk Assessment Division, National Institute of Food and Drug Safety Evaluation,

Cheongju 28159, Republic of Korea

^ǂ^ These authors contributed equally on this work

**^*^C*orresponding authors***:

**Chang-Beom Park, Ph. D.** (Principal Researcher),

Environmental Exposure & Toxicology Research Center, Korea Institute of Toxicology (KIT), Jinju 52834, Republic of Korea. Tel: +82-55-750-3735; E-mail: [changbeom.park@kitox.re.kr](mailto:changbeom.park@kitox.re.kr)

^[[1]](#footnote-1)^

**Table S1**. Primer sequences used for sequencing of adult zebrafish samples via quantitative real-time polymerase chain reaction (qRT-PCR). Target genes: aryl hydrocarbon receptor (*ahr2*), cytochrome P450 subunits (*cyp1a1* and *cyp1b1*), nuclear factor erythroid 2-related factors (*nrf1a* and *nrf2a*), proto-oncogene (*p53*), thyroid stimulating hormone beta (*tsh-β*), thyroid hormone receptor alpha and beta (*thraa* and *thrb*), and housekeeping gene (*β-actin*).

| **Biological response** | **Gene name**  **(Gene No.)** | **Primer** | **Sequences (5'–3')** | **Ref.** |
| --- | --- | --- | --- | --- |
| Chemical stimulation | *ahr2*  (NM_131264.1) | Forward | GGGAAGGTGGTTCTTGGCTAC | Ren et al., 2020 |
|  |  | Reverse | CTCCTGTCTTTATCATTCTGATGTGGTT |  |
|  | *cyp1a1*  (NM_131879.2) | Forward | GCATTACGATACGTTCGATAAGGAC |  |
|  |  | Reverse | GCTCCGAATAGGTCATTGACGAT |  |
|  | *cyp1b1*  (NM_001045256.1) | Forward | AGTGTGTTGCTGTCGCTGATG |  |
|  |  | Reverse | GAGAACGGACCCGGTACCA |  |
| Cell damage | *nrf1a*  (JX867114) | Forward | CCAGAGTTGACAGGTCCTGG | Williams et al., 2013 |
|  |  | Reverse | CATAACCTGTGATTCCATGATAGAC |  |
|  | *nrf2a*  (JX867116) | Forward | GAGCGGGAGAAATCACACAGAATG |  |
|  |  | Reverse | CAGGAGCTGCATGCACTCATCG |  |
|  | *p53*  (ID: 30590) | Forward | CAGGCCCATCCTCACAAT | Fan et al., 2018 |
|  |  | Reverse | TTCCTTCGTCCTTCACCA |  |
| Developmental hormones | *tsh-β*  (AY135147) | Forward | GCAGATCCTCACTTCACCTACC | Liu et al., 2011 |
|  |  | Reverse | GCACAGGTTTGGAGCATCTCA |  |
|  | *thraa*  (NM_131396) | Forward | CTATGAACAGCACATCCGACAAG |  |
|  |  | Reverse | CACACCACACACGGCTCATC |  |
|  | *thrb*  (NM_131340) | Forward | ATCGACCAGAGCCCACACA |  |
|  |  | Reverse | TAGGTGCCGATCCAATGTCTT |  |
| Housekeeping | *β-actin*  (AF057040.1) | Forward | ACCCACACCGTGCCCATCTA | Liu et al., 2011 |
|  |  | Reverse | CGGACAATTTCTCTTTCGGCTG |  |

**Table S2**. Relative fold change of gene expression in zebrafish embryos exposed to clothianidin (CLO), imidacloprid (IMD) and, their mixture at different concentrations, at 6 days after exposure. All data represent the mean value ± standard error (SEM) (n = 15 embryos in each group with triplicate experiments). ^a, b^Different letters indicate significant difference between the exposure groups (*P* < 0.05). Mix. 1, 10, and 100 indicate binary mixtures of CLO and IMD, each at 1, 10, and 100 ng/mL.

| Target genes | Relative fold change of gene expression in each exposure group | | | | | |
| --- | --- | --- | --- | --- | --- | --- |
|  | Single chemical (ng/mL) | | | Binary mixtures at a 1 : 1 combined ratio of CLO and IMD (ng/mL) | | |
|  | Cont. (sol) | CLO 10 | IMD 10 | Mix. 1 | Mix. 10 | Mix. 100 |
| *ahr2* | 1.08±0.32^a^ | 0.24±0.04^a^ | 0.48±0.32^a^ | 6.57±1.10^b^ | 7.74±2.70^b^ | 6.85±2.94^b^ |
| *cyp1a1* | 1.03±0.18^a^ | 0.38±0.06^a^ | 1.20±0.80^a^ | 17.90±3.94^b^ | 11.54±0.39^b^ | 12.84±1.54^b^ |
| *cyp1b1* | 1.08±0.32^a^ | 1.36±0.34^a^ | 2.28±1.24^a^ | 9.51±1.24^b^ | 11.02±0.66^b^ | 8.20±1.09^b^ |
| *nrf1a* | 1.27±0.54^a^ | 0.12±0.03^a^ | 0.10±0.03^a^ | 12.74±2.29^b^ | 14.62±4.03^b^ | 13.01±2.40^b^ |
| *nrf2a* | 1.11±0.37^a^ | 0.32±0.04^a^ | 0.26±0.05^a^ | 8.19±2.27^b^ | 6.61±2.17^b^ | 5.98±0.40^b^ |
| *p53* | 1.06±0.26^a^ | 0.004±0.001^b^ | 0.007±0.001^b^ | 0.006±0.001^b^ | 0.008±0.002^b^ | 0.009±0.001^b^ |
| *tsh-β* | 1.02±0.14^a^ | 0.31±0.01^b^ | 0.26±0.07^b^ | 0.27±0.04^b^ | 0.27±0.04^b^ | 0.39±0.11^b^ |
| *thraa* | 1.09±0.34^a^ | 0.28±0.07^b^ | 0.34±0.05^b^ | 0.35±0.09^b^ | 0.26±0.02^b^ | 0.33±0.04^b^ |
| *thrb* | 1.04±0.22^a^ | 0.14±0.02^b^ | 0.20±0.02^b^ | 0.17±0.03^b^ | 0.15±0.02^b^ | 0.16±0.01^b^ |

**Figure S1.** Concentration-response relationships of clothianidian (A) and imidacloprid (B) for zebrafish embryo-larva. All data are shown as the mean ± standard error of mean. The fitted sigmoidal dose-response curve represented by the Hill 3-parameter equation (SigmaPlot version 12.5, Systat Software, USA).

1. Abbreviations: *ahr2*, Aryl hydrocarbon receptor; *cyp1a1* and *cyp1b1*, cytochrome P450 subunits; CLO, clothianidin; IMD, imidacloprid; *nAChRs*, nicotinic acetylcholine receptors; *neonics*, neonicotinoid pesticides; *nrf1a* and *nrf2a*, nuclear factor erythroid 2-related factor; *p53*, proto-oncogene; *tsh-β*, thyroid stimulating hormone beta; *thraa* and *thrb*, thyroid hormone receptor alpha and beta [↑](#footnote-ref-1)
