# Supplementary material for: Cocktail effects of clothianidin and imidacloprid in zebrafish embryonic development, with high and low concentrations of mixtures
Source: Front Toxicol. 2024 Sep 18;6:1464069. doi: 10.3389/ftox.2024.1464069 (PMC11445189; doi:10.3389/ftox.2024.1464069)
Supplement: Supplementary file 2 [file Image1.pdf]

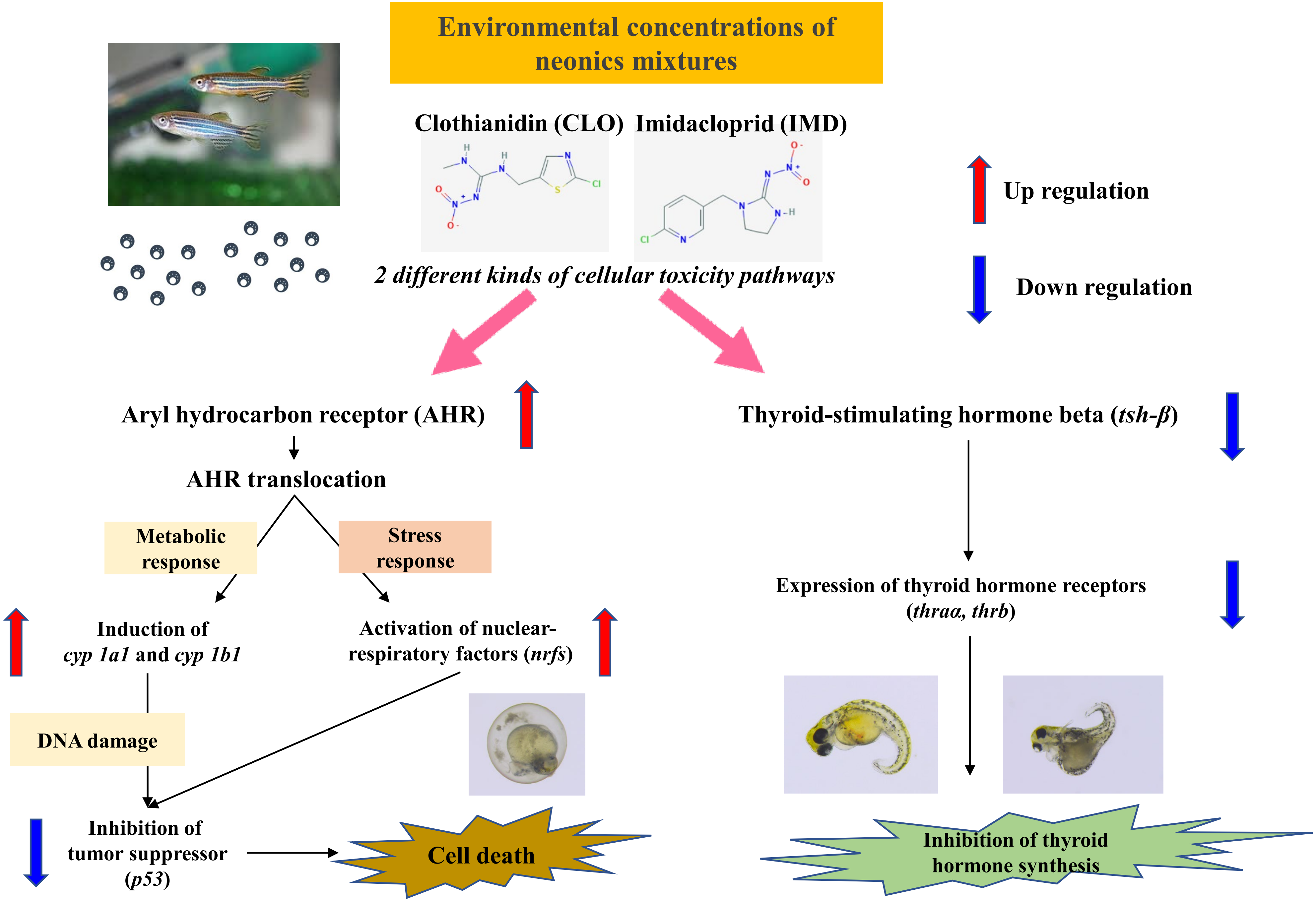

Environmental concentrations of neonics mixtures
Clothianidin (CLO)
Imidacloprid (IMD)
2 different kinds of cellular toxicity pathways
Up regulation
Down regulation
Aryl hydrocarbon receptor (AHR)
AHR translocation
Metabolic response
Stress response
Induction of cyp 1a1 and cyp 1b1
Activation of nuclear-respiratory factors (nrfs)
DNA damage
Inhibition of tumor suppressor (p53)
Cell death
Thyroid-stimulating hormone beta (tsh-β)
Expression of thyroid hormone receptors (thraα, thrb)
Inhibition of thyroid hormone synthesis
